# Neuropsychological decrements in midlife type-2 diabetes are not associated with peripheral NLRP3 inflammasome responsiveness

**DOI:** 10.3389/fimmu.2022.1021351

**Published:** 2022-10-13

**Authors:** Adam H. Dyer, Isabella Batten, Conor Reddy, Liam Townsend, Conor P. Woods, Desmond O’Neill, James Gibney, Sean P. Kennelly, Nollaig M. Bourke

**Affiliations:** ^1^ Age-Related Healthcare, Tallaght University Hospital, Dublin, Ireland; ^2^ Inflammaging Research Group, Trinity Translational Medicine Institute, Dublin, Ireland; ^3^ Department of Medical Gerontology, School of Medicine, Trinity College Dublin, Dublin, Ireland; ^4^ Wellcome-HRB Clinical Research Facility, St. James’s Hospital, Dublin, Ireland; ^5^ Department of Infectious Diseases, St. James’s Hospital, Dublin, Ireland; ^6^ Robert Graves Institute of Endocrinology, Tallaght University Hospital, Dublin, Ireland

**Keywords:** diabetes, cognition, dementia, inflammation, midlife

## Abstract

Midlife Type 2 Diabetes Mellitus (T2DM) is associated with an increased risk of Alzheimer Disease (AD) in later life, with altered inflammatory responses postulated as key pathological drivers. Previous studies have demonstrated increased responsiveness to NLR family pyrin domain containing 3 (NLRP3) inflammasome agonists, both in individuals with untreated T2DM in addition to those with established AD. We hypothesised that peripheral NLRP3 inflammasome responses may be altered during the early stages of T2DM-related cognitive dysfunction. Here, we assessed the relationship between NLPR3 responses in peripheral blood mononuclear cells (including to Aβ-42, the putative pathogenic protein in AD) and neuropsychological performance in uncomplicated midlife T2DM to identify early signatures of immune dysregulation which may predispose to later cognitive decline. We recruited a cross-sectional cohort of middle-aged adults with uncomplicated T2DM and matched Healthy Controls (HCs) for comprehensive neuropsychological assessment and *in vitro* PBMC responses to a range of NLRP3 agonists were assessed. T2DM was associated with subtle decrements on neuropsychological tests of delayed memory and executive function (both p<0.05). Overall, there were no differences between T2DM and HCs in immune responses induced by NLRP3 agonists. Further, we observed no relationship between the subtle neuropsychological decrements observed in T2DM and PBMC responsiveness to NLRP3 agonists. Our data suggests that peripheral NLRP3 inflammasome response dysregulation may not play a role in the early stages of cognitive dysfunction in midlife T2DM. Further longitudinal studies are warranted to examine the contribution of peripheral NLRP3 responses towards disease pathology and as cognitive decline accelerates in T2DM.

## Introduction

Type 2 Diabetes Mellitus (T2DM) is a common metabolic disease strongly linked to accelerated cognitive decline, particularly in midlife (typically during the 4^th^-6^th^ decades of life), where it is associated with a greater risk of Alzheimer Disease (AD) in later life (1, 2). In fact T2DM may be as strong a risk factor for AD as *APOE* genotype, the greatest genetic risk factor for sporadic AD (3). Evidence suggests that the risk of AD in midlife T2DM is linked to the duration of T2DM, with earlier age of T2DM onset associated with a greater AD risk (4). Thus, there may be an important ‘critical window’ in preventing later cognitive decline and AD in midlife T2DM. The efficacy of potential preventative multi-domain interventions, shown to reduce dementia incidence in high-risk populations, have typically not been studied in midlife T2DM (5–7). There is an urgent need to understand which individuals with midlife T2DM are at greatest risk of later cognitive decline in order to select-out those at greatest risk for potential preventative interventions, as well as gain an understanding of the pathological drivers linked with cognitive decrements early in disease development.

Hypotheses linking midlife T2DM and AD include the role of hyper (and hypo) -glycaemia, brain insulin resistance, micro/macrovascular pathology and impaired amyloid clearance by microglial cells within the brain (8). Central to many of these mechanisms is the key role of inflammation. T2DM is associated with a pro-inflammatory state, with activation of Pathogen Recognition Receptors (PRRs) by Damage Associated Molecular Patterns (DAMPs) such as Islet Amyloid Polypeptide (IAPP) and Free Fatty Acids (FFAs) (9, 10). Inflammation initially localised to the pancreas in T2DM later results in low-grade systemic inflammation, detected as an elevated serum levels of pro-inflammatory cytokines (11). Such a pro-inflammatory state may serve to “prime” or “skew” subsequent immune responses to various DAMPs, such as amyloid beta (Aβ), a putative pathogenic protein in AD.

Recently, the importance of the NLR family pyrin domain containing 3 (NLRP3) inflammasome, an intracellular PRR that detects and responds to a variety of exogenous and endogenous stimuli, has been recognised in the pathogenesis of T2DM. Activation of NLRP3 results in binding to apoptosis associated speck-like protein containing a CARD (ASC), which in turn interacts with caspase-1 to form the inflammasome complex (“priming” step or “signal 1”). Activation of caspase-1 results in the cleavage and subsequent maturation of IL-1β from pro-IL-1β - an inflammasome-dependent cytokine (“activation step” or “signal 2”) (12). In T2DM, NLRP3 can become activated by IAPP/FFAs amongst other DAMPs, resulting in release of the pro-inflammatory inflammasome-dependent cytokine IL-1β (13). Notably, Peripheral Blood Mononuclear Cells (PBMCs) in individuals with treatment-naïve T2DM have demonstrated a heightened state of NLRP3 activation in comparison to healthy controls in response to DAMPS including FFA and IAPP (14). Yet despite strong evidence that NLPR3 associated responses may be linked with the detrimental inflammatory characteristics of this disease, whether this NLRP3 hyper-responsiveness seen in T2DM may be related to the risk of cognitive decline and AD in T2DM, has not been explored.

Deposition of Aβ plaques is one of the pathophysiological hallmarks of AD, the most common form of sporadic dementia (15). Initially, a great deal of research focussed on the direct impact of these plaques on neuronal function, however in recent years it has emerged that the immune response elicited by pathogenic Aβ may be of central importance in the pathogenesis of AD (16–18). Importantly, the brain’s resident macrophages, microglia, express NLRP3 and Aβ deposition in the brain is capable of activating the NLRP3 inflammasome (19). This has even led to interest in NLRP3 inhibitors for the treatment of AD (20–22). As with studies above for T2DM, a heightened state of NLRP3 activation in PBMCs from individuals with established AD has been demonstrated (23). Indeed, interest has now moved towards examining peripheral signatures of NLRP3 activation in individuals at increased risk of AD, free from established cognitive impairment in order to identify those at greater risk of later cognitive decline.

For instance, an emerging body of literature has focussed on identifying individuals without established cognitive impairment or dementia who have subtle cognitive decrements on neuropsychological testing. PBMCs from individuals with IQ-discrepant neuropsychological performance show a heightened response to LPS, an important primer of the NLRP3 inflammasome, in addition to a heightened inflammatory response to DAMPS such as LPS & Aβ (24, 25). Whilst previous studies have examined the association between circulating pro-inflammatory cytokines and cognitive dysfunction in older adults with T2DM (26–28), no study has examined the relationship between NLRP3 responsiveness and cognitive function in T2DM, which may represent an important common pathway mediating AD risk in midlife T2DM.

Here, we examined the relationship between midlife T2DM and *in vitro* PBMC responses to NLRP3 inflammasome agonists (including Aβ). We hypothesised that T2DM would be associated with a heightened inflammatory response to NLRP3 agonists that may be associated with neuropsychological performance in midlife T2DM. Our aim was to evaluate whether peripheral immune responses would correlate with detailed assessments of neuropsychological function in individuals with uncomplicated T2DM in midlife in order to potentially identify those who may be at greatest risk of later cognitive decline.

## Methods

### Participant recruitment and health assessment

The current study was embedded within the ENBIND (Exploring Novel Biomarkers of Brain Health in Type 2 Diabetes) study (28, 29). Ethical Approval was obtained from the Tallaght-St James’s Joint Research Ethics Committee (Reference: 2018/09/02/2018-10 List 34(4)). Briefly, ENBIND aimed to investigate the impact of uncomplicated midlife Type 2 Diabetes Mellitus (T2DM) on neuropsychological function to identify individuals with midlife T2DM who may be at greater risk of later cognitive decline. Participants were recruited from T2DM clinics along with age and sex matched Healthy Controls (HC) by local advertisement in a 2:1 ratio. Participants were 35-65 years of age without a diagnosis of cognitive impairment, significant Diagnostic and Statistics Manual (DSM) Axis I psychiatric illness or neurological disorder. Participants were free from significant musculoskeletal, cardiac or respiratory comorbidity. Individuals on long-term immunosuppressive medication or with a history of autoimmune disease were excluded. For the T2DM group, participants were free from screened macrovascular (previous stroke, myocardial infarction, ischaemic heart disease or peripheral vascular disease) or microvascular (diabetic retinopathy, peripheral neuropathy or diabetic nephropathy) complications.

Detailed information on medical history was collected in addition to any history of hypertension (prescribed hypertensive medication of a seated clinic blood pressure of ≥140/90 mmHg) or hypercholesterolaemia (prescribed lipid-lowering therapy or total cholesterol/low density lipoprotein above local reference range).

### Neuropsychological assessment

All participants were assessed in the first instance using the Montreal Cognitive Assessment (MoCA) as a test of global cognitive performance and had a score of 24/30 or greater for participation in order to exclude those with potential established cognitive impairment from detailed neuropsychological testing (30). Detailed neuropsychological testing was carried out using the Cambridge Neuropsychological Assessment Battery (CANTAB™) (31, 32). A custom-designed battery was used for the current study, which consisted of 70 minutes of detailed neuropsychological assessment across 5 tasks. For each task, scores were transformed to Z-scores across the study population to aid in analysis and interpretability. Tasks consisted of:

i. **Paired Associates Learning**: memorising geographical locations of patterns on the screen
ii. **Spatial Working Memory (SWM):** memorising the locations of tokens on screen and remembering their location
iii. **Pattern Recognition Memory Delayed (PRMD):** memorising patterns and asked to recall after a 20 minute delay
iv. **One-Touch Stockings of Cambridge (OTS):** matching patterns by moving coloured balls inside stockings in the minimum number of moves
v. **Rapid Visual Processing (RVP):** detecting sequences of numbers amongst a rapidly changing span of digits.

All testing was conducted by the same physician trained in neuropsychological assessment.

### PBMC preparation and stimulation

Blood samples were obtained from participants after screening assessment and prior to detailed neuropsychological testing. Briefly, 27mL of blood was collected in lithium heparin vacutainers™ and transported to the research laboratory for same-day preparation of PBMCs performed using a density-gradient separation medium (Histopaque-1077). Fresh PBMCs were plated at a density of 1 x 10^6^ cells per mL of media (containing RPMI supplemented with 10% FBS and 1% Penicillin-Streptomycin) and incubated at 37°C under the following conditions for 18 hours:

**i. Unstimulated:** incubated in media only for 18 hours
**ii. Lipopolysaccharide (LPS):** 100ng/mL LPS added for 18 hours to assess innate immune activation *via* Toll-Like Receptor 4 (TLR4) agonism - an important “primer” of NLRP3 inflammasome responses
**iii. Amyloid β-42 (Aβ-42):** 10nM Aβ-42 added for 18 hours – the putative pathogenic amyloid implicated in AD and known to activate NLRP3
**iv. LPS & Aβ-42 Combined**: 100ng/mL LPS & 10nM Aβ-42 – to evaluate Aβ-42 induced NLRP3 activation in LPS “primed” PBMCs
**v. LPS & Nigericin**: 100ng/mL LPS for 18 hours with 1μM Nigericin added for the final 2 hours. Nigericin is a potent activator of the NLRP3 inflammasome as was used as a model of “maximal” NLRP3 agonism in LPS-primed PBMCs

Following incubation for 18 hours, supernatant was harvested and remaining cells re-suspended in a fresh solution of lysis buffer (PureLink RNA lysis buffer) for storage at -20°C and -80°C respectively for future RNA extraction and qPCR analysis.

### RNA extraction and quantitative polymerase chain reaction analysis

RNA was extracted from PBMC samples using a PureLink mini-RNA extraction kit (Termo-Scientific™) and RNA quantified using a NanoDrop spectrophotometer to measure RNA concentration and assess RNA purity. All RNA samples were treated with DNase (DNase I, Amplification Grade Kit; Thermo-Scientific) and cDNA transcribed using the High Capacity cDNA Reverse Transcription Kit (Thermo Scientific) following the protocol provided within the respective kits. For all RNA samples, 250ng of high-quality RNA was reverse transcribed into cDNA. qPCR was conducted using Sybr green reagent (Thermo-Scientific) and analysed using QuantStudio 5.0 software. The 2^-ΔΔCT^ method was used to express the relative concentration of each gene of interest in comparison to the housekeeping gene 18S. Primers (IDT Technologies) were designed to be non-intron spanning and of at least 200 bps in length using the NCBI Primer BLAST database (See **Table S1**)

### Enzyme-Linked Immunosorbent Assays (ELISA)

ELISA kits were used as per manufacturer’s instructions to quantify the quantity of the inflammasome dependent cytokine IL-1β (R&D Systems DY201) in addition to IL-6 (BD Biosciences BD 555220) in cell supernatant and read using a plate-reader at 450nm.

### Statistical analysis

Statistical analysis was carried out using GraphPad Prism v9.0.1 and STATA v17.0. Data were first assessed for normality by inspecting histograms and Shapiro-Wilk tests where appropriate. Descriptive statistics are presented as means (with standard deviations) and medians (with interquartile ranges) for parametric and non-parametric data respectively. Between group differences (T2DM vs HCs) were assessed using t-tests and Wilcoxon rank-sum tests as appropriate. Paired analysis was conducted using Wilcoxon sign-rank tests for non-parametric data. In order to analyse the potential correlation between protein production/gene expression and neuropsychological variables, Spearman correlation was used, with results analysed in the cohort as a whole regardless of T2DM status, followed by stratified analysis for those with midlife T2DM and HCs. For correlational analysis, a Bonferonni test for multiple comparisons was applied.

## Results

### Study cohort

Overall, 58 middle-aged participants were recruited (median age: 53, 47-59 years; 31/58; 53.5% female). Those with T2DM (n = 38) had a significantly greater BMI (p = 0.008) and greater burden of hypertension (p < 0.001) and hypercholesterolaemia (p < 0.001) than the HC (n = 20) group. There were no significant differences between T2DM and HCs in age, sex or years of formal education. Those with T2DM had a median duration of T2DM of 5 years (IQR: 2-10 years). Most (28/38; 73.7%) were prescribed regular metformin, either alone (n = 8) or in combination with other oral anti-diabetic medication (n = 28). One-fifth (n = 8) used a subcutaneous Glucagon Like Peptide (GLP-1) analogue in combination with an oral agent. Two participants were untreated at time of study recruitment. Overall, those with T2DM had a median HbA1c of 54mmol/mol (IQR: 44-81). No participants in the HC group had a HbA1c in the diabetic range. Detailed characteristics by study group are given in **Table 1**.

**Table 1:** Cohort Characteristics: A Cohort of individuals with uncomplicated T2DM free from any macrovascular complications in addition to a cohort of matched HCs were recruited.

Characteristic	HC (n = 20)	T2DM (n = 38)	Statistic
Age (years), *median* (IQR)	51.5 (47-60.5)	53.5 (46-57)	p = 0.77
Sex (female), n (%)	13 (65%)	18 (47.4%)	p = 0.20
Body Mass Index, *median* (IQR)	26.8 (24.8-28.4)	30 (27.5-35)	**p = 0.01**
Educational Attainment			
*Primary, n (%)* *Secondary, n (%)* *Tertiary, n (%)*	1 (5%) 12 (60%) 7 (35%)	5 (13.2%) 25 (65.8%) 8 (21.1%)	p = 0.39
Family History of Dementia, n (%)	3 (15.8%)	7 (18.4%)	p = 0.81
Hypertension, n (%)	2 (10%)	25 (65.8%)	**p < 0.001**
Hyperlipidaemia, n (%)	2 (10.5%)	26 (68.4%)	**p < 0.001**
Charlson Comorbidity Index, *median* (IQR)	1 (0-2)	2 (1-3)	**p = 0.01**
HbA1c (mmol/mol), *median* (IQR)	37.5 (34-38)	54 (44-81)	**p < 0.001**
C-Reactive Protein (mg/dL), *median* (IQR)	1 (0-2)	1 (1-3.5)	p = 0.22
***Cognitive & Neuropsychological Assessment***			
MoCA Score, *median* (IQR)	29 (28-30)	29 (27-30)	p = 0.11
Paired Associates Learning (Z-Score), mean (6)	-0.04 (0.16)	-0.65 (0.96)	p = 0.06
Spatial Working Memory (Z-Score), mean (6)	-0.26 (1.21)	0.14 (0.86)	p = 0.08
Pattern Recognition Memory, Delayed (Z-Score), mean (6)	0.38 (1.03)	-0.20 (0.94)	**p = 0.03**
One Touch Stockings of Cambridge (Z-Score), mean (6)	0.31 (1.10)	-0.16 (0.92)	**p = 0.04**
Rapid Visual Processing (Z-Score), mean (6)	0.03 (1.11)	-0.02 (0.95)	p = 0.43

A summary of background demographic characteristics in addition to clinical characteristics is shown. Individuals with midlife T2DM had a higher BMI in addition to a greater burden of hypertension and hypercholesterolaemia. T2DM was associated with poorer performance on neuropsychological tests of delayed memory (Pattern Recognition Memory, Delayed) and executive function (One Touch Stockings of Cambridge). IQR, Interquartile Range; SD, Standard Deviation; T2DM, Type 2 Diabetes; HC, Healthy Controls. Bold values refer to statistically significant results (p<0.05).

### Subtle decrements in neuropsychological tests of delayed memory and executive function in midlife T2DM

Participants underwent detailed neuropsychological assessment in order to assess for early signs of cognitive dysfunction in T2DM and correlate these with inflammatory responses. Global cognitive performance (assessed using the MoCA) did not significantly differ between HC and midlife T2DM. Overall, participants with midlife T2DM had significantly poorer performance on two of the detailed neuropsychological tests: (i) Pattern Recognition Memory – Delayed (PRMD), a test of delayed memory and (ii) One Touch Stockings of Cambridge (OTS), a neuropsychological test with significant executive function demands (**Table 1**) (both p<0.05). There were trends for poorer performance on tests of working memory in those with T2DM, but these differences were not statistically significant (**Table 1**).

### Baseline expression of pro-inflammatory and NLRP3 associated genes

**Figure 1:**
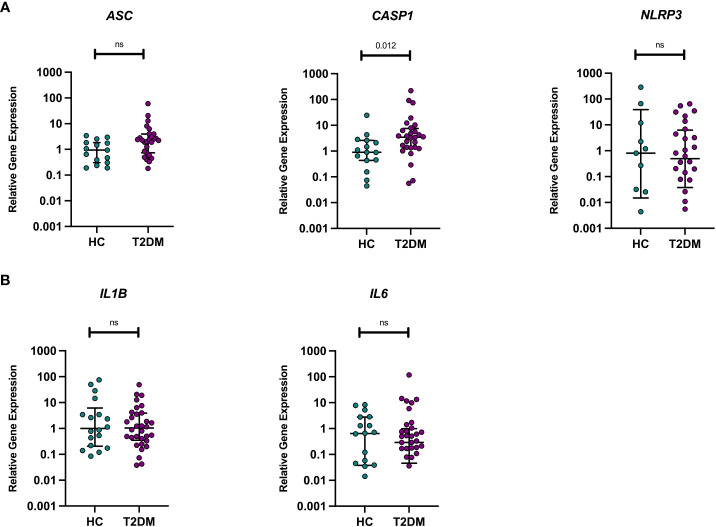
Unstimulated pro-inflammatory and NLRP3 inflammasome genes in T2DM and HCs. Differences in baseline gene expression (from unstimulated PBMCs) were assessed between individuals with midlife T2DM (n = 31) and HCs (n = 19). qPCR was used to quantify gene expression and data are presented normalised to the median of healthy control sample panel **(A)**. There were no differences in the transcription levels of *ASC or NLRP3*, whilst those with midlife T2DM had significantly greater *CASP* expression in comparison to HCs (p = 0.012, wilcoxon rank-sum test) panel **(B)**. There were no differences in the transcription levels of IL1B or IL-6 between midlife T2DM and HCs. Data are presented as individual data points, with median and interquartile range indicated by black lines. Statistical analysis was conducted using wilcoxon rank-sum tests. T2DM, Type 2 Diabetes; HC, Healthy Controls; n, numbers.

Given the known heightened inflammatory state in T2DM and the potential role of NLRP3 in mediating later AD risk in midlife T2DM, we assessed differences in baseline gene expression between those with midlife T2DM and HCs in (i) pro-inflammatory (*IL1B, IL6*) and (ii) NLRP3-inflammasome related genes (*NLRP3, ASC, CASP1*). No significant differences in gene expression were observed between HC and T2DM samples with the exception of *CASP1* gene expression levels, with significantly higher transcript in unstimulated PBMCs from those with T2DM (p<0.05, **Figure 1**).

### Immune responses to NLRP3 agonists are not altered in uncomplicated midlife T2DM

Given the association between T2DM in midlife and later cognitive decline, and in line with evidence demonstrating heightened PBMC NLRP3 immune responses in both T2DM and AD, we examined whether PBMC inflammatory responses to stimulation with Aβ-42 and other NLRP3 agonists were altered in midlife T2DM.

**Figure 2:**
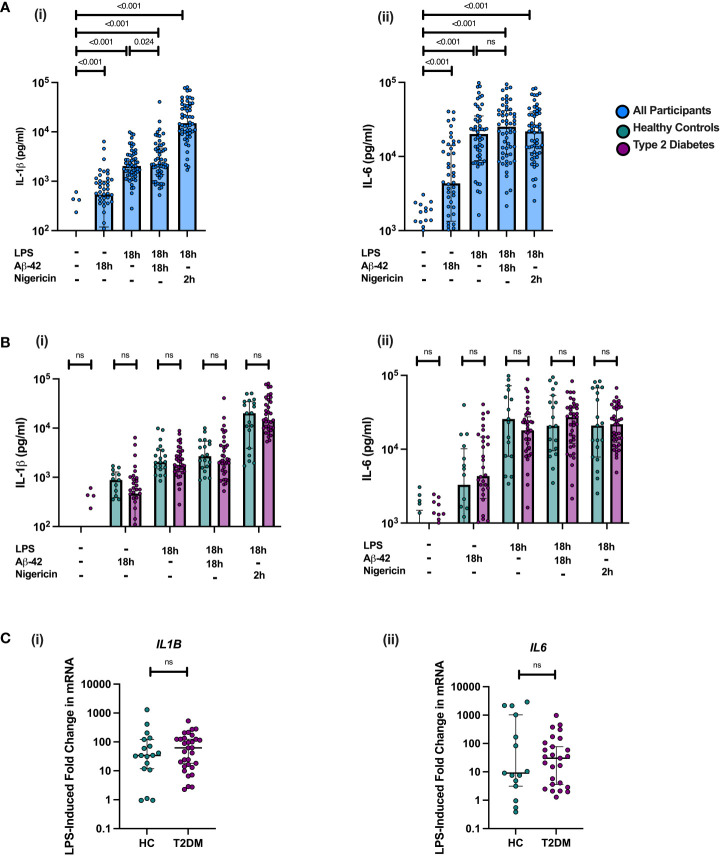
PBMC responses to NLRP3 Agonists are not altered in Midlife T2DM. PBMCs were isolated from individuals with midlife T2DM (n = 38) and matched HCs (n = 20) and stimulated with Amyloid Beta (Aβ-42), both alone and in combination with LPS. PBMCs were also incubated with LPS and nigericin to assess robust NLRP3 inflammasome responses. Cytokine production is following incubation is presented in pg/mL in supernatant and gene expression calculated as fold change relative to unstimulated control for each sample. Data are presented as individual data points, with median and interquartile range. **(A)** Treatment with Aβ-42, both alone and in combination with LPS resulted in a significant production of both IL-1β **(i)** and IL-6 **(ii)** by PBMCs (all p<0.001, Wilcoxon matched-pairs test). Greater IL-1β release was seen in under the Aβ-42 + LPS condition in comparison to LPS alone (p = 0.024, wilcoxon matched-pairs), which was not seen for IL-6 concentration. **(B)** There were no significant differences in protein production between T2DM and HC in responses to any of the agonists studied. **(C)** Fold change in both *IL1B* and *IL6* mRNA following LPS treatment did not differ between T2DM and HCs. Statistical analysis was performed using Wilcoxon matched-pairs tests for matched data; ns, non-significant. T2DM, Type 2 Diabetes; HC, Healthy Control; h, hours; n, numbers.

Treatment of PBMCs with LPS alone for 18 hours resulted in a significant pro-inflammatory response in PBMCs (IL-1β and IL-6 cytokine production in comparison to unstimulated condition) (all p<0.001, **Figure 2**). This response did not significantly differ between individuals with midlife T2DM and HCs (**Figure 2**). Incubation of PBMCs with Aβ-42 alone induced a strong pro-inflammatory response measured *via* IL-6 and IL-1β production (all p<0.001, **Figure 2**). However, this response did not differ between midlife T2DM and HCs (**Figure 2A**)

In order to assess the impact of Aβ-42 on NLPR3 activation in “primed” immune cells, PBMCs were incubated for 18 hours with a combination of Aβ-42 and LPS. Consistent with NLRP3 inflammasome activation, this combination resulted in a significantly greater cytokine production of IL-1β (an inflammasome dependent cytokine) but not IL-6 in PBMCs than for LPS alone on paired analysis (p<0.05, **Figure 2**). There was no difference between T2DM and HCs in IL-1β or IL-6 production for PBMCs incubated with the combination of LPS and Aβ-42 (**Figure 1A**)

Further, we examined responses to the combination of LPS and Nigericin, a known potent activator of the NLRP3 inflammasome in order to assess maximal NLRP3 inflammasome activation. Incubation with LPS for 18 hours and Nigericin added for the final 2 hours resulted in a much greater release of IL-1β than any of the Aβ-42 and LPS conditions (all p<0.001, **Figure 2B**). This robust effect was not seen for IL-6, consistent with NLRP3 activation (**Figure 2B**). There were no differences between midlife T2DM and HCs in LPS and Nigericin induced IL-1β production (**Figure 2B**).

In examining the transcriptional response to LPS in stimulated PBMCs, *IL6* and *IL1B* gene expression was significantly increased in following incubation for 18h (p<0.001; **Figure 2**), but the fold change in mRNA expression (compared to unstimulated PBMC expression) did not differ between T2DM and HCs. Addition of Aβ-42 to LPS-primed PBMCs for 18 hours did not result in significantly increased gene expression of *IL6* or *IL1B* than LPS alone, consistent with a priming effect of LPS on PBMCs exposed to Aβ-42 (**Figure S1**).

On correlational analysis, there was no relationship between baseline (unstimulated) expression of NLPR3-assocaited genes (NLRP3, ASC, CASP1) and NLPR3-dependent cytokine production for any of the four PBMC stimulation conditions, either in individuals with T2DM, HCs or the combined cohort (**Table S2**).

### Neuropsychological decrements in T2DM are not associated with peripheral NLRP3 responses

**Figure 3:**
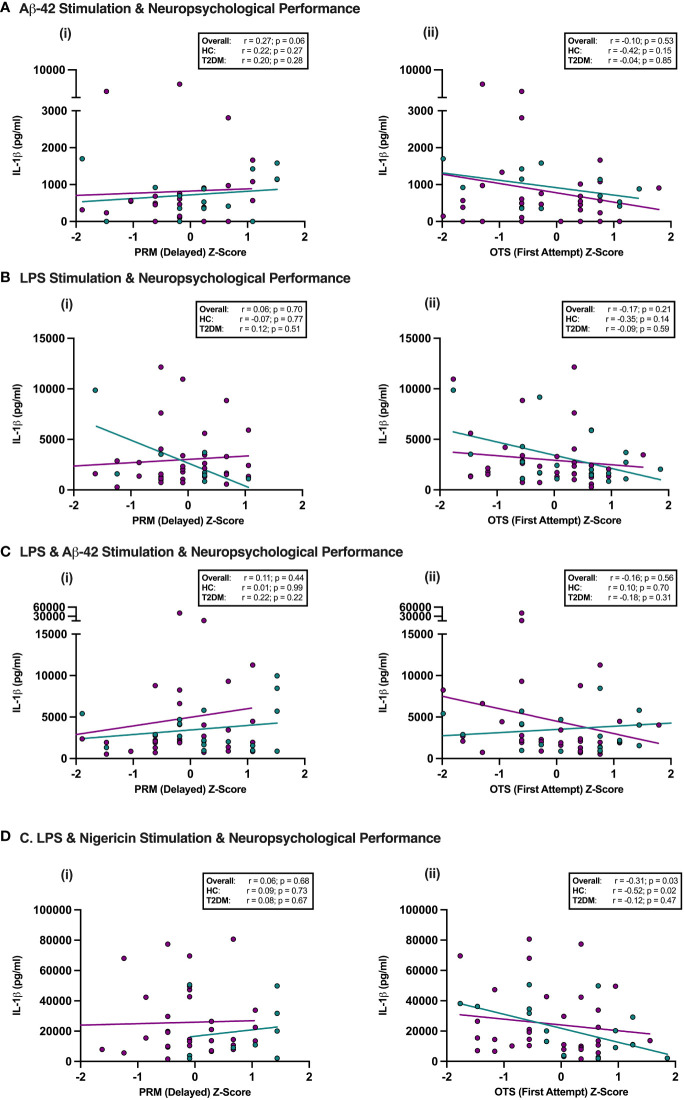
NLRP3 responses in Midlife T2DM and Neuropsychological performance. Participants underwent detailed assessment of neuropsychological function using a custom-designed assessment battery of five-tests including Pattern Recognition Memory (PRM), a test of delayed memory and One Touch Stockings of Cambridge (OTS), a test with significant executive function demands. PBMCs were isolated and stimulated with Aβ-42 alone, LPS alone, LPS + Aβ-42 or LPS + Nigericin, and the relationship between NLRP3-induced cytokine production (IL-1β) and neuropsychological performance in individuals with and without T2DM assessed. **(A–D)** The only significant association observed was between better performance on executive function and lesser maximal IL-1β release on LPS + Nigericin stimulation in the overall cohort and in HCs, which did not persist on Bonferonni correction for multiple testing. PRM, Pattern Recognition Memory; OTS, One Touch Stockings of Cambridge; HC, Healthy Controls; T2DM, Type 2 Diabetes.

Given the potential role of NLRP3 in mediating the link between T2DM and later cognitive decline, we tested whether there was any relationship between the subtle neuropsychological decrements seen in midlife T2DM and peripheral NLRP3 inflammasome responses. Overall, there was no association between performance on either tests of delayed memory (Pattern Recognition Memory Delayed) or executive function (One Touch Stockings of Cambridge) and NLRP3 agonist induced PBMC responses (measured *via* IL-1β release, an inflammasome dependent cytokine) in T2DM (**Figure 3**). On detailed correlational analysis of all five neuropsychological tests, no significant relationships between immune cell responses to any of the four stimulation conditions and neuropsychological performance were observed following adjustment for multiple testing (**Table S3**).

## Discussion

The current study aimed to assess whether T2DM in midlife, the time-period when T2DM is acting as a risk factor for cognitive dysfunction, was associated with altered PBMC immune responses to various NLRP3 agonists including Aβ-42, the putative pathogenic protein in AD. T2DM was not associated with a heightened immune responsiveness to Aβ-42 in our study, measured *via* production of pro-inflammatory cytokines. Whilst we demonstrated subtle (but significant) decrements in performance in both delayed memory and executive function tasks on detailed neuropsychological assessment in midlife T2DM, these decrements were not associated with NLRP3 responsiveness. This data provides key insights into the early pathophysiological effects in T2DM and associated cognitive decline and rule out peripheral NLRP3 responses as being detectably involved at this early stage.

In our study, Aβ-42, both in the presence and absence of LPS, induced a strong pro-inflammatory response. Predictably, IL-1β production in response to stimulation with Aβ-42 & LPS in combination was significantly greater than either LPS and Aβ-42 alone. However, treatment with Aβ-42 & LPS did not induce a greater transcriptional response than LPS alone, supporting a NLRP3 priming role for LPS and a subsequent activation role of Aβ-42. Notably, these stimulations were far less potent than the NLRP3 agonist nigericin, used in our experiments as a model of maximal NLRP3 activation and subsequent IL-1β production. Importantly, response to either of the four stimulation conditions (LPS alone, Aβ-42 alone, LPS & Aβ-42, LPS & Nigericin) did not differ between individuals with T2DM in midlife and HC. These responses were unrelated to the subtle neuropsychological decrements seen in those with T2DM.

These results are surprising given previous evidence demonstrating altered PBMC responsiveness to various DAMPs in both T2DM and AD. However, it is important to remember that the cohort under study in the current analysis were cognitively normal, free from any subjective or objective cognitive impairment and had a median MoCA score (median 29/30 in both groups) above even what would be expected from population-based studies (30). This is reflective of the design of our current study, which sought to assess the earliest possible indicators of the potential relationship between NLPR3-related immune dysregulation and cognitive function in midlife T2DM. It is very possible that the population under study were too cognitively healthy to demonstrate any pathological relationship between immune responses and cognitive dysfunction, given the subtle nature of the cognitive decrements in this cognitively-healthy population.

Further, individuals with T2DM in the current study were all on treatment (except for two individuals) and treatment with metformin and other oral T2DM medication are known to have an important anti-inflammatory effect (33–35). Additionally, GLP-1 analogues, used by a significant minority of individuals in the current study are known to exert potent anti-inflammatory effects and are even being investigated as potential treatment for sporadic AD (36–38). Thus, by design the fact that nearly all of our participants were established on treatment for T2DM may have introduced an important immunomodulatory effect into the T2DM group. Importantly, in previous studies examining altered PBMC responses to inflammatory stimuli, T2DM participants were not on treatment and these elevated innate immune responses significantly improved following treatment with metformin, at which point they did not differ from the healthy control group (14). In this context, our findings are in agreement with previous literature for PBMC immune responses in individuals with treated T2DM.

Additionally, we studied a very young population with a high level of education and minimal other comorbidities. By design, our study excluded individuals with significant medical comorbidity in addition to those with any micro or macro-vascular complications of diabetes. Whilst previous evidence has demonstrated important associations between age-at-onset in T2DM and subsequent cognitive decline, it may be many years before such decline becomes evident (4). Overall, our study participants had a relatively short duration of diabetes, and well controlled diabetes, which may have influenced our results. However these exclusions allowed us to examine the relationship between these responses without such potential confounding effects and therefore strengthen the conclusions of our data. Importantly, further naturalistic studies using more a more heterogenous cohort are needed to determine when exactly T2DM may be exhibiting a pro-inflammatory state pre-disposing to later cognitive, which may aid in selecting-out those at greater risk of later cognitive decline.

Despite recruiting a healthy population, we observed significant decrements in executive function and memory performance in midlife T2DM, that were not associated with IL-1β and IL-6 production in response to several pro-inflammatory agonists, possibly indicating that these individuals are in the earliest stages of cognitive dysfunction. Future work, both in the ENBIND cohort and other studies, should therefore seek to examine the longitudinal relationships between cognitive decrements and inflammatory responses in order to identify potential windows of susceptibility for potential preventative interventions. Whilst no studies have examined the relationship between peripheral immune cell responses and cognition in T2DM longitudinally, recent evidence from the Edinburgh Type 2 Diabetes study suggests that serum cytokine concentration can predict cognitive decline at 10-year follow up in T2DM (39). Longitudinal studies examining the relationship between immune-response dysregulation and the development of cognitive decline in T2DM are warranted, and may be able to select-out those with midlife T2DM at greatest risk of later cognitive decline.

Our study has several important limitations that are worthy of discussion. In the first instance, we included individuals with T2DM free from complications and established on treatment with minimal other comorbidity. This very specific population may not be representative of the wider population with T2DM and may have minimised differences between T2DM and HC participants. However, this population were purposefully collected in the current study in order to assess the earliest indicators of the peripheral inflammation-cognitive dysfunction link in T2DM. Further, our study is cross-sectional in nature and only includes a single time-point. By design we were unable to examine longitudinal changes in immune cell responses and neuropsychological performance.

Finally, it is important to reflect that our analysis of PBMC responses to Aβ-42 and other NLRP3 agonists is of unknown relevance to microglial responses to Aβ-42 – still a major unanswered question in the field. By analysing peripheral responses, we were unable to infer the inflammatory state of the Central Nervous System (CNS). Future studies using more direct assessments of CNS inflammation (such as studies of cerebrospinal fluid or indeed imaging of microglial activation states) may offer important insight into the relationship between peripheral inflammation and CNS inflammation to determine if T2DM is associated with an “inflammatory resonance” between the periphery and CNS. Shedding new light on this relationship could lead to the establishment of novel biomarkers which capture a potential pro-inflammatory state, which may be an important target for preventative and anti-inflammatory intervention in midlife T2DM.

## Conclusion

In conclusion, we assessed PBMC responses to a variety of DAMPs (including Aβ-42, the putative pathogenic protein in AD) in midlife T2DM. We found no differences between T2DM and HC PBMC responsiveness to these DAMPs, which was not explained by baseline levels of pro-inflammatory or NLRP3 inflammasome gene transcripts. There was no relationship between peripheral NLRP3 responses and cognitive function in midlife T2DM, even though individuals with T2DM in midlife demonstrated significantly poorer performance on tests of delayed memory and executive function. Our data therefore suggests no role for peripheral NLRP3 responses in the early stages of T2DM-associated cognitive decline. Future studies should focus on longitudinal assessment of immune cell responses and cognitive function, in order to select-out individuals at greatest risk of future cognitive decline.

## Data availability statement

The original contributions presented in the study are included in the article/Supplementary Materials. Further inquiries can be directed to the corresponding author.

## Ethics statement

The studies involving human participants were reviewed and approved by Tallaght-St James’s Joint Research Ethics Committee [Reference: 2018/09/02/2018-10 List 34(4)]. The patients/participants provided their written informed consent to participate in this study.

## Author contributions

AD, SK, and NB designed the protocol, conducted the study and had oversight on recruitment, assessment and analysis of laboratory parameters. IB, LT, and CR assisted with laboratory experiments. CW and JG assisted with participant recruitment. SK, CW, DO’N, NB, and AD assisted with concept and design of the study in addition to critically appraising the final manuscript. AD, NB, and SK: paper writing and revision. All authors approved the final manuscript.

## Funding

The ENBIND Study was funded by a grant from the Meath Foundation. This work was performed within the Irish Clinical Academic Training (ICAT) Programme, supported by the Wellcome Trust and the Health Research Board (Grant Number 203930/B/16/Z), the Health Service Executive, National Doctors Training and Planning, and the Health and Social Care, Research and Development Division, Northern Ireland. AHD was additionally supported by the Wellcome-HRB Clinical Research Facility, St James’s Hospital, Dublin, Ireland.

## Acknowledgments

The authors wish to acknowledge all study participants in addition to staff from the Department of Age-Related Healthcare, Tallaght University Hospital, and The Robert Graves Institute of Endocrinology, Tallaght University Hospital

## Conflict of interest

The authors declare that the research was conducted in the absence of any commercial or financial relationships that could be construed as a potential conflict of interest.

## Publisher’s note

All claims expressed in this article are solely those of the authors and do not necessarily represent those of their affiliated organizations, or those of the publisher, the editors and the reviewers. Any product that may be evaluated in this article, or claim that may be made by its manufacturer, is not guaranteed or endorsed by the publisher.

## Supplementary material

Supplementary Figure 1 There was no significant difference in the transcription of *IL1B* or *IL6* in cells stimulated with LPS alone and cell stimulated with LPS and Aβ-42; ns: not-significant
