# Supplementary material for: Neuropsychological decrements in midlife type-2 diabetes are not associated with peripheral NLRP3 inflammasome responsiveness
Source: Front Immunol. 2022 Oct 13;13:1021351. doi: 10.3389/fimmu.2022.1021351 (PMC9607906; doi:10.3389/fimmu.2022.1021351)
Supplement: Supplementary Figure 1 — There was no significant difference in the transcription of IL1B or IL6 in cells stimulated with LPS alone and cell stimulated with LPS and Aβ-42; ns: not-significant [file DataSheet_1.docx]

**Supplementary Materials**

**Table S1**

| **Gene** | **Forward Sequence** | **Revers Sequence** |
| --- | --- | --- |
| ***18S*** | GTAACCCGTTGAACCCCATT | CCATCCAATCGGTAGTAGCG |
| ***IL1B*** | TCGCCAGTGAAATGATGGCT | TGGAAGGAGCACTTCATCTGTT |
| ***IL6*** | CCTTCTCCACAAACATGTAACAAGA | TCACCAGGCAAGTCTCCTCA |
| ***ASC*** | CCGGGATCCAAGGTGAACAA | CAGTCTTGAAGGTCCGGAGG |
| ***CASP1*** | GCCTGTTCCTGTGATGTGGA | TTCACTTCCTGCCCACAGAC |
| ***NLRP3*** | CTTTCTGTGTGGACCGAAGC | AGCCCTTCTGGGGAGGATAG |

**Table S1. Primer Sequences;** Forward and reverse primers were designed using the NCBI BLAST database and were non-intron spanning and of at least 200 bps in length.

**Figure S1**


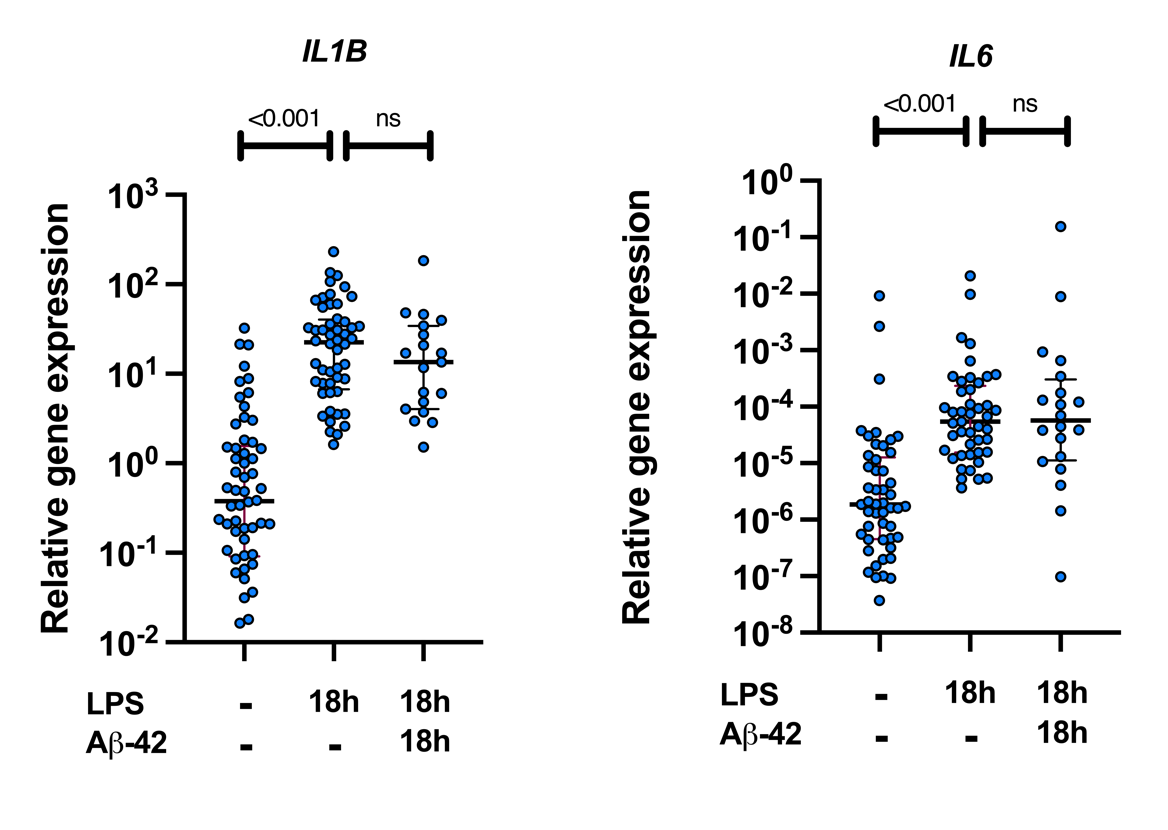


**Figure S1**. There was no significant difference in the transcription of *IL1B* or *IL6* in cells stimulated with LPS alone and cell stimulated with LPS and Aβ-42; ns: not-significant

**Table S2**

|  | **ASC** | **CASP** | **NLRP3** |
| --- | --- | --- | --- |
| **IL1β Release** |  |  |  |
| ***Overall Cohort*** |  |  |  |
| Unstimulated | -0.08 | -0.02 | -0.18 |
| AB42 | 0.01 | -0.17 | -0.12 |
| LPS | -0.04 | -0.04 | 0.15 |
| LPS & AB42 | -0.04 | -0.07 | 0.07 |
| LPS & Nigericin | 0.21 | 0.06 | 0.41 |
| ***T2DM Only*** |  |  |  |
| Unstimulated | -0.20 | -0.21 | -0.21 |
| AB42 | 0.29 | 0.07 | -0.06 |
| LPS | 0.03 | 0.09 | 0.21 |
| LPS & AB42 | -0.05 | -0.01 | 0.12 |
| LPS & Nigericin | 0.38 | 0.09 | -0.11 |
| ***Controls Only*** |  |  |  |
| Unstimulated | -0.31 | -0.03 | 0.63 |
| AB42 | -0.42 | -0.46 | -0.38 |
| LPS | -0.11 | -0.02 | 0.20 |
| LPS & AB42 | -0.16 | -0.06 | 0.16 |
| LPS & Nigericin | -0.32 | -0.06 | 0.63 |

**Table S2** **Correlation Analysis between Inflammasome Gene Transcripts and release of the inflammasome-dependent pro-inflammatory cytokine, IL-1β**. Results are presented for the overall cohort and separately in those with T2DM and HCs. Analysis was performed using Spearman correlation analysis and coefficients in the above table represent spearman rho values. No correlations were statistically significant

**Table S3**

|  | **PAL** | **SWM** | **PRMD** | **OTS** | **RVP** |
| --- | --- | --- | --- | --- | --- |
| ***Overall Cohort*** |  |  |  |  |  |
| *IL-1β Release* |  |  |  |  |  |
| Unstimulated | -0.24 | 0.16 | -0.01 | -0.08 | -0.15 |
| AB42 | -0.01 | -0.04 | 0.27 | -0.10 | 0.09 |
| LPS | 0.01 | 0.19 | 0.06 | -0.17 | 0.09 |
| LPS & AB42 | -0.00 | 0.06 | 0.11 | -0.16 | 0.05 |
| LPS & Nigericin | -0.07 | -0.02 | 0.06 | -0.31 | -0.06 |
| *IL6 Release* |  |  |  |  |  |
| Unstimulated | 0.25 | 0.01 | 0.06 | 0.23 | 0.18 |
| AB42 | 0.17 | -0.19 | 0.28 | -0.07 | -0.08 |
| LPS | 0.05 | 0.16 | -0.07 | 0.08 | 0.02 |
| LPS & AB42 | 0.21 | 0.13 | 0.13 | 0.14 | -0.04 |
| LPS & Nigericin | 0.08 | 0.28 | -0.02 | -0.13 | 0.06 |
| ***T2DM Only*** |  |  |  |  |  |
| *IL-1β Release* |  |  |  |  |  |
| Unstimulated | -0.18 | 0.16 | 0.07 | -0.17 | -0.10 |
| AB42 | -0.01 | 0.03 | 0.20 | -0.04 | 0.16 |
| LPS | 0.02 | -0.01 | 0.12 | -0.09 | 0.18 |
| LPS & AB42 | -0.03 | -0.15 | 0.22 | -0.18 | 0.17 |
| LPS & Nigericin | -0.01 | -0.16 | 0.08 | -0.12 | -0.15 |
| *IL6 Release* |  |  |  |  |  |
| Unstimulated | 0.17 | 0.03 | -0.01 | 0.10 | 0.19 |
| AB42 | 0.06 | -0.34 | 0.38 | -0.14 | -0.05 |
| LPS | 0.16 | -0.18 | -0.14 | 0.11 | -0.10 |
| LPS & AB42 | 0.18 | -0.09 | 0.11 | 0.17 | -0.18 |
| LPS & Nigericin | 0.35 | -0.10 | 0.74 | -0.03 | 0.01 |
| ***HC Only*** |  |  |  |  |  |
| *IL1B release* |  |  |  |  |  |
| Unstimulated | -0.35 | 0.11 | -0.05 | 0.30 | -0.31 |
| AB42 | -0.29 | -0.04 | 0.22 | -0.42 | 0.01 |
| LPS | -0.03 | 0.52* | -0.07 | -0.35 | -0.06 |
| LPS & AB42 | 0.15 | 0.52* | 0.01 | 0.10 | -0.21 |
| LPS & Nigericin | -0.05 | 0.20 | 0.09 | -0.52* | 0.04 |
| *IL6 Release* |  |  |  |  |  |
| Unstimulated | 0.20 | 0.25 | -0.15 | 0.30 | 0.26 |
| AB42 | 0.48 | -0.10 | 0.41 | 0.13 | -0.10 |
| LPS | -0.14 | 0.08 | -0.11 | -0.03 | 0.25 |
| LPS & AB42 | 0.31 | 0.04 | 0.06 | 0.10 | 0.14 |
| LPS & Nigericin | -0.13 | 0.38 | -0.05 | -0.12 | -0.08 |

**Table S3**. **No relationship between other cognitive tests and PBMC responses**. *p<0.05. No significant associations persisted on Bonferroni correction for multiple comparisons. PAL: paired associates learning; SWMS: Spatial Working Memory Score; PRM: Pattern Recognition Memory (Delayed); OTS: One Touch Stockings of Cambridge; RVP: Rapid Visual Processing.
